# Editorial: New perspectives on the role of sensory feedback in speech production, volume II

**DOI:** 10.3389/fnhum.2026.1838493

**Published:** 2026-04-29

**Authors:** John F. Houde, Lucie Ménard, Jeffery A. Jones, Douglas M. Shiller, Xing Tian

**Affiliations:** 1 Department of Otolaryngology – Head and Neck Surgery, University of California, San Francisco, San Francisco, CA, United States; 2 Département de Linguistique, Université du Québec à Montréal, Montréal, QC, Canada; 3 Department of Psychology, Wilfrid Laurier University, Waterloo, ON, Canada; 4 École d'orthophonie et d'audiologie, Université de Montréal, Montréal, QC, Canada; 5 Department of Neural and Cognitive Sciences, New York University Shanghai, Shanghai, China; 6 New York University-East China Normal University Institute of Brain and Cognitive Science at New York University Shanghai, Shanghai, China; 7 Shanghai Key Laboratory of Brain Functional Genomics (Ministry of Education), School of Psychology and Cognitive Science, East China Normal University, Shanghai, China

**Keywords:** auditory feedback, sensory feedback, somatosensory feedback, speech perception, speech production

This Research Topic, focusing on the role of sensory feedback in speech production, is meant to be inclusive to all sense modalities. However, most prior studies on this topic have focused on auditory feedback, and there are at least two reasons for this emphasis. First, conceptually, speaking primarily involves producing sound sequences that have meaning to the listener, and so it seems natural that auditory feedback, which allows the speaker to hear what their listeners hear, would be the most influential feedback modality. Second, pragmatically, as pointed out by Casserly and Marino in this volume, feedback alteration studies are very direct ways of determining sensory feedback's role in speaking and are much easier to carry out in the auditory domain than in somatosensation—the other main source of feedback for a speaker.

Nevertheless, as shown by the studies of Bourhis et al. and Casserly and Marino in this volume, there are ways of investigating some of the effects of non-auditory modalities on speaking.

The Original Research article by Bourhis et al. is entirely devoted to examining somatosensory feedback perturbation responses. In their study, sudden, small forward yanks of the tongue during the whispered production of a vowel were quickly compensated for, even when subjects were prevented from hearing the whispered auditory feedback by masking noise. This study adds more evidence to the idea that speech goals, while initially being represented in auditory features alone, are for most speakers also represented in terms of somatosensory features. And indeed, a prior study suggested that once participants have both auditory and somatosensory representations of a speech sound, it is a matter of individual sensory weighting as to which sense modality is relied on more to guide production (Lametti et al., 2012).

The Original Research article by Casserly and Marino shows that topical benzocaine numbs somatosensation at the lips without appreciably affecting lip muscles and that speech produced under combined degraded somatosensory and auditory feedback is less intelligible than under degraded auditory feedback alone. This result is consistent with prior studies suggesting that speaking relies on both auditory and somatosensory feedback. But what about visual feedback? Casserly and Marino found that allowing speakers to see their face in a mirror did nothing to improve their intelligibility under degraded auditory and somatosensory feedback. In fact, when auditory and somatosensory feedback were left unaltered, allowing speakers to see their mirror image degraded intelligibility. This result suggests a crucial lesson: for a sensory feedback modality to be constructive for speech production, the speaker needs to have accumulated sensorimotor experience with that feedback modality while speaking. The McGurk effect (McGurk and MacDonald, 1976) shows we have intersensory associations from both seeing and hearing others' speech, but not many of us have spent much time watching ourselves speak in front of a mirror. Without such sensorimotor experience, the study by Casserly and Marino suggests the initial effects of the new sense modality may be destructive.

Another article in this volume is a Brief Research Report by Subrahmanya et al. on a phenomenon referred to as pitch centering, which occurs across repetitions of a voiced utterance. Each utterance has a pitch that initially deviates from the median pitch, but this deviation tends to reduce as production of the utterance progresses. This results in a reduction in variance (across repetitions of a voiced utterance) from initial pitch to the pitch at a later point in the utterance—in this case, after about 150 ms. The study not only examined this phenomenon in healthy, normal speakers but also looked at how this pitch-centering phenomenon differs between healthy controls and patients with Alzheimer's disease (AD). AD patients showed an asymmetry in the amount of centering observed when initial pitch was lower than the median vs. when initial pitch was higher than the median.

The centering phenomenon seen in both the control of pitch as well as formants seems to be evidence for online feedback control being active in normal speech and not just as a reaction to an artificial change in sensory feedback created in the laboratory. Centering shows that initial deviations from what is likely the intended target (which the median of a number of repetitions of the utterance is likely to represent) are rapidly detected and used by the speech motor control system to generate corrections during the utterance. But what sensory modality is likely to be used to detect these initial deviations? Prior studies have shown that the addition of masking noise to what the subject is hearing does not reduce or change the degree of centering produced by a subject. This fact, along with the observation that centering seems to begin within milliseconds of utterance onset, suggests that it is likely not dominated by auditory feedback but rather is mostly controlled by somatosensory feedback.

To conclude, let us consider the Hypothesis Article authored by Usler regarding the nature of somatosensory feedback. Usler proposes an explanation of stuttering behavior based on the Active Inference model of sensory processing and motor control. This model blurs the line between the sensory goals of a movement and the predicted sensory feedback. Usler proposes that stuttering results largely from abnormal precision and timing of sensory feedback, which interferes with the action-perception cycle that forms the foundation of movement control. While Usler discusses sensory feedback abstractly throughout most of the paper, the model hypothesizes that the lowest levels of movement control rely on equilibrium point control of the vocal tract articulators, which in turn is highly dependent on somatosensory/proprioceptive feedback from proprioceptors in muscle fibers and tendon organs.

Considered from this point of view, and indeed regardless of the specific motor control model chosen, all movements rely fundamentally on a feedback control loop between alpha motor neurons, the muscle fibers they innervate, and the gamma intrafusal fibers that sense changes in muscle length (Kandel et al., 2000). Thus, at this lowest level of speech motor control, information about muscle fiber state is sensed by feedback proprioceptive signals originating from within the muscle groups and tendons that produce articulatory forces. In other words, at the lowest level of movement control (the motor unit), all sensory feedback is effectively somatosensory in nature.

Collectively, the articles in this volume challenge the auditory-centric view of speech motor control. These studies demonstrate that somatosensory feedback is not secondary to auditory feedback but is instead integral to speech production, from the rapid compensation for articulatory errors to the fundamental feedback loops of the motor unit.

## References

[B1] Kandel E. R. Schwartz J. H. Jessell T. M. (2000). Principles of Neural Science, 4th Edn. New York, NY: McGraw-Hill.

[B2] Lametti D. R. Nasir S. M. Ostry D. J. (2012). Sensory preference in speech production revealed by simultaneous alteration of auditory and somatosensory feedback. J. Neurosci. 32, 9351–9358. doi: 10.1523/JNEUROSCI.0404-12.2012 22764242 PMC3404292

[B3] McGurk H. MacDonald J. (1976). Hearing lips and seeing voices. Nature 264, 746–748. doi: 10.1038/264746a0 1012311

